# Additive Manufactured Polymers in Dentistry, Current State-of-the-Art and Future Perspectives-A Review

**DOI:** 10.3390/polym14173658

**Published:** 2022-09-03

**Authors:** Codruta Victoria Tigmeanu, Lavinia Cosmina Ardelean, Laura-Cristina Rusu, Meda-Lavinia Negrutiu

**Affiliations:** 1 Department of Technology of Materials and Devices in Dental Medicine, Faculty of Dental Medicine, Multidisciplinary Center for Research, Evaluation, Diagnosis and Therapies in Oral Medicine, “Victor Babes” University of Medicine and Pharmacy Timisoara, 2 Eftimie Murgu Sq., 300041 Timisoara, Romania; 2 Department of Oral Pathology, Faculty of Dental Medicine, Multidisciplinary Center for Research, Evaluation, Diagnosis and Therapies in Oral Medicine, “Victor Babes” University of Medicine and Pharmacy Timisoara, 2 Eftimie Murgu Sq., 300041 Timisoara, Romania; 3 Department of Prostheses Technology and Dental Materials, Faculty of Dental Medicine, Research Center in Dental Medicine Using Conventional and Alternative Technologies, “Victor Babes” University of Medicine and Pharmacy Timisoara, 2 Eftimie Murgu Sq., 300041 Timisoara, Romania

**Keywords:** 3D printing, additive manufacturing, 4D printing, polymers, bioinks, bioprinting, scaffolds, tissue engineering, digital dentistry

## Abstract

3D-printing application in dentistry not only enables the manufacture of patient-specific devices and tissue constructs, but also allows mass customization, as well as digital workflow, with predictable lower cost and rapid turnaround times. 4D printing also shows a good impact in dentistry, as it can produce dynamic and adaptable materials, which have proven effective in the oral environment, under its continuously changing thermal and humidity conditions. It is expected to further boost the research into producing a whole tooth, capable to harmoniously integrate with the surrounding periodontium, which represents the ultimate goal of tissue engineering in dentistry. Because of their high versatility associated with the wide variety of available materials, additive manufacturing in dentistry predominantly targets the production of polymeric constructs. The aim of this narrative review is to catch a glimpse of the current state-of-the-art of additive manufacturing in dentistry, and the future perspectives of this modern technology, focusing on the specific polymeric materials.

## 1. Introduction

Polymer-based materials play an important role in dentistry, with a wide variety of applications, based on their surface characteristics, mechanical and biological properties, easy processing, and affordable cost [1].

Some of the most commonly used polymers in dentistry are polymethyl methacrylate (PMMA), polyurethane (PU), polyethylene (PE), polycarbonate (PC), polyetheretherketone (PEEK), polyethylene glycol (PEG), polydimethylsiloxane (PDMS), polylactic acid (PLA), poly(e-caprolactone) (PCL), acrylonitrile butadiene styrene (ABS), polypropylene (PP) [2].

Their mechanical properties are related to the bulk material characteristics, but the interaction with oral tissues is highly dependent on their surface, justifying the use of polymer coatings to increase their biocompatibility [3,4]. The specific applications of polymers cover almost every field of dentistry, including direct restorative procedures, prosthodontics, orthodontics, and even implantology, as synthetic PEEK has been recently proposed as an implant material [5,6]. Customized polymeric facial prostheses, with detailed morphology, can be easily obtained by 3D printing. Polymers have also been used to manufacture scaffolds, with role in regeneration of bone structures, dentin- and pulp-like tissues, membranes for guided tissue regeneration, and as drug delivery systems in treating numerous oral and periodontal pathologies [7].

Composite resins for direct restorations are probably the most used polymers in dentistry, in association with dental adhesives, which, besides bonding the restorations to the dental surface, play an important role in the adhesion of brackets, retainers, and bands in orthodontics. Moreover, fiber-reinforced composites are used for post-orthodontic tooth retention and for splinting mobile teeth. Various types of polymers are widely used in prosthodontics, as well, including denture manufacturing, and various other types of prosthodontic appliances, as adhesives for metal or ceramic, veneering materials etc., [1,2].

The continuous development of their applications and manufacturing technologies in prosthodontics, including injection and CAD/CAM milling, has finally resulted in a most versatile technique, namely 3D printing.

3D printing, also known as additive manufacturing or rapid prototyping, was first used in 1980s [8,9]. Due to the development of the printing techniques and devices, it currently allows a wide range of applications, medicine included. 3D printing has recently become in the forefront of research in biomedical fields, as it enables the manufacture of patient specific devices and tissue constructs [10].

**Figure 1:**
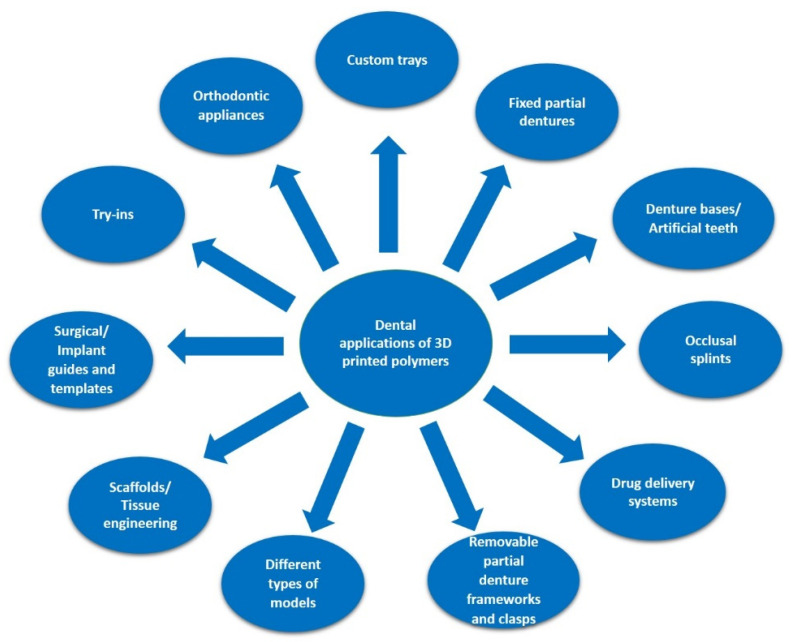
Indications of 3D-printed polymers in dentistry.

This manufacturing technology is currently used in a large variety of medical and biomedical applications including anatomical or experimental models, medical devices, prostheses and implants, scaffolds, tissues and organs, anatomical structures, and drug delivery systems [11,12,13]. Vat photo-polymerization, droplet-based, extrusion-based, and powder-based 3D-printing techniques have already proven their importance for modern dentistry, covering a wide range of indications (Figure 1) [14].

Different types of materials, including polymers, metals, and ceramics, are used for 3D printing in dentistry. However, because of their high versatility associated with the wide variety of available materials, 3D printing in dentistry predominantly targets the production of polymeric constructs [15].

3D bioprinting uses so-called “bioinks” to fabricate complex organ structures and functional tissues that can support live cells and other biological factors [16]. By means of 3D bioprinting, tailored tissue-engineered constructs, with defined structures and properties can be speedily manufactured, including cells, DNA, growth factors, and other bioactive components as integral parts of the building process [16].

Currently, there is a growing trend to develop both novel, high-quality 3D printable biomaterials and bioinks with specific properties and high printability.

The aim of this narrative review is to catch a glimpse of the current state-of-the-art of additive manufacturing in dentistry, and the future perspectives of this modern technology, focusing on the specific polymeric materials.

The following databases: PubMed, MEDLINE, and Web of Science, were electronically searched for articles reporting the use of 3D-printed polymers in dentistry, using a combination of multiple keywords including “dentistry” OR “digital dentistry”, AND “polymers” OR “bioinks”, AND “3D printing” OR “4D printing” OR “additive manufacturing” OR “3D bioprinting”. Only full-text articles, written in English have been considered. A further manual search has been carried out, based on the relevant cited references.

## 2. 3D Printing Technologies in Dentistry

The 3D-printing technologies used for dental purposes show noticeable differences in the technology, as well as in resolution, accuracy, and repeatability [17] (Table 1).

Currently, the most widespread additive technology in dentistry is vat photo-polymerization, including the stereolithography (SLA) and digital light processing (DLP) methods. The principle is based on liquid photopolymer in a vat being selectively cured by light-activated polymerization [18]. In SLA, the polymerization is performed by a directed UV-laser beam, used to sequentially cure liquid photopolymer resin layers, while in DLP, a whole layer of photosensitive liquid resin is simultaneously polymerized by a UV-light mask. SLA is characterized by high resolution and good accuracy, being suitable for fine details and functional prototyping, while DLP is characterized by high printing resolution, fast production rate, and affordable costs [19].

**Figure 2:**
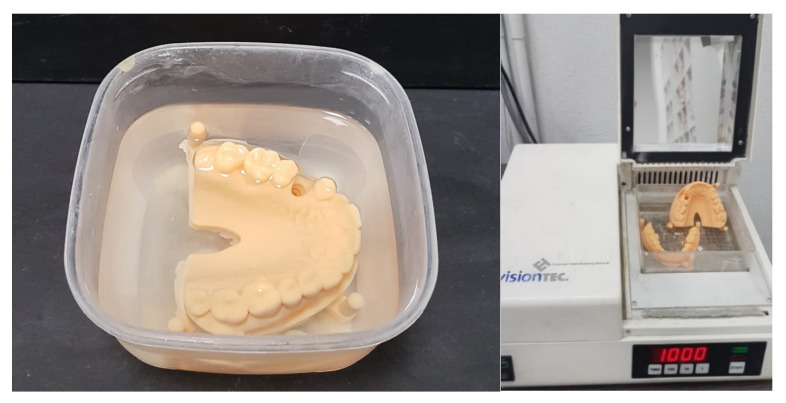
3D vat photo-polymerized models, cleaned with isopropanol, and post-polymerized.

Regardless of the method, the printed parts need subsequent cleaning with isopropanol, to remove the excessive monomer, and post-polymerization with UV-light (Figure 2) [10,18]. Both SLA and DLP are versatile techniques as they can be used with a wide variety of polymeric materials [20].

Continuous direct light processing (CDLP), based on the continuous liquid interface production (CLIP) technology, which was patented in 2015, involves a continuous high speed build process, and high object precision, suitable for denture bases and bite splints [21]. The additional use of a membrane allows oxygen permeation, and inhibits radical polymerization [20].

Material jetting (MJT) or photo-polymer jetting involves applying tiny drops of material directly to the build platform via the print head, followed by photo-polymerization in an intermediate exposure step, and requires no post-polymerization. The object is built on a layer by layer basis, extremely fast and highly accurate. A special feature is the multi-material 3D-printing multicolor mode [21].

Unlike vat photo-polymerization and MJT, which use photo-polymeric materials, fusion deposition modelling (FDM) is an extrusion-based printing technique in which melted thermoplastic materials are being used to manufacture the desired object, using layer by layer deposition. Initially used only for polymeric structures, it has been subsequently modified to process ceramics and composites [22]. Despite the low cost, because of the longer printing times and lower resolutions, FDM is currently considered of lesser relevance for dental purposes [21]. A wide range of polymeric materials, in a filament form, have been considered: ABS, PC, thermoplastics, polyamide (PA), polystyrene (PS), polyetherimide (PEI) and polyoxymethylene (POM) as well as polyethylene (PE) [23].

Selective laser sintering (SLS), which uses a high-power pulsed laser to fuse polymeric, metallic or ceramic powder particles, by creating surface layers, is currently mostly used in dentistry for printing metallic structures [10].

Binder jetting (BJ) utilizes selectively deposited liquid bonding agents to fuse powdered material [20].

**Figure 3:**
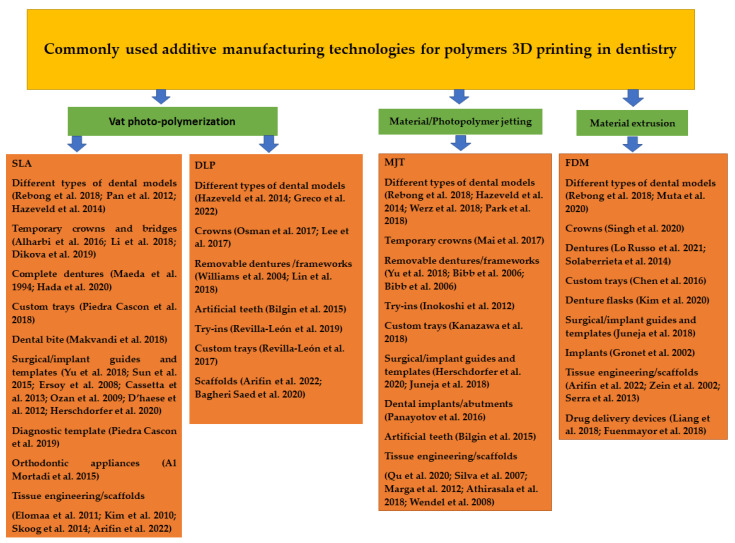
Most used additive manufacturing technologies and their applications for polymers 3D printing in dentistry [24,25,26,27,28,29,30,31,32,33,34,35,36,37,38,39,40,41,42,43,44,45,46,47,48,49,50,51,52,53,54,55,56,57,58,59,60,61,62,63,64,65,66,67,68,69,70,71,72,73,74,75,76,77,78,79,80].

The applications of most commonly used additive manufacturing technologies in dentistry are presented in Figure 3.

## 3. 3D Bioprinting Technologies for Dental and Maxillo-Facial Applications

3D-printing technologies have demonstrated great potential in the fabrication of delicate but diversified structures for biomedical applications [81]. 3D bioprinting is currently an essential technique for fabricating scaffolds for tissue engineering purposes, which aim to restore the physiological and histological characteristics of the injured tissue [82].

In 3D printing, “printability generally refers to the ability of a material to be fabricated in a layer-by-layer sequence into a 3D object with well controlled design” [16]. For biomedical applications, further criteria are needed, since the constructs are intended to host and maintain cell proliferation. Bioprinting must encompass the reproducibility, structural integrity, and fidelity of typical 3D printing as well as certain requirements regarding compatibility with living organisms: non-toxicity, biodegradability, adhesion to cells, and porosity [16].

Several bioprinting strategies have been explored so far [83], extrusion-based bioprinting being considered the most applicable for tissue engineering, due to its ease in printing bioactive bioinks [84]. Extrusion-based technique requires thixotropic bioinks, such as fluids like hydrogels, showing reducing viscosity under applied shear stress, as the ejected filament-like structures are larger than the droplet sizes [85,86]. Extrusion-based bioprinting enables printing of highly viscous cell-loaded bioinks by adjusting the air pressure during the pneumatic deposition [87], but, unfortunately, the applied extrusion pressure exposes the cells to a noticeable level of stress which can affect the cell viability [88,89]. A recent approach to the increment of FDM use is the inclusion of electrospinning [90].

Other 3D bioprinting methods, including SLA, inkjet, laser, have been increasingly used in tissue engineering applications recently, but, due to their complex functionality, they incur higher costs, as compared to the extrusion technique [91].

SLA is a highly tunable bioprinting technique, which uses photosensitive bioinks. The photo-crosslinking light source enables printing various patterns in a layer-by-layer manner, from a bioink reservoir onto a movable platform [92]. One of its advantages is that it can utilize high-viscosity bioinks, with minimum mechanical stresses on the encapsulated cells. The typically mechanical stresses generated by more viscous hydrogels are being avoided, resulting in higher cell viability and functionality [93].

SLA and DLP enable high-speed construction of complex, heterogeneous, and accurate architecture, including various medical implants and tissue models. The structures and properties of the 3D-printed scaffolds can be tailored according to the practical requirements, mimicking the heterogeneity and complexity of natural tissues, by selecting the appropriate bioink combinations. The types and concentrations of photoinitiators and crosslinkers, exposure time and intensity can also be modulated [81].

Inkjet bioprinting is a relatively fast method that deposits small ink droplets into a predetermined location, driven by thermal or piezoelectric actuation. It offers high-resolution printing of single cells [94]. When using inkjet bioprinting, the material needs to show a time-dependent increase in viscosity, thus allowing the droplet formation after ejection. Thus, only bioinks with certain values of viscosity may be used [95,96].

The laser-based methods enable relatively high resolution printing of biological material such as cells, but due to the use of a pulsed laser source, their viability can be compromised [97,98].

Laser-induced forward transfer (LIFT) uses a pulsed laser as an energy source to induce a jet formation to the substrate, and requires a bioink with a determined viscosity range [97]. Due to the relatively low viscosity, the 3D structure needs appropriate cross-linking post printing, to improve its mechanical integrity [97].

SLS has also been attempted for fabricating tissue-engineering scaffolds, but with limitations due to low retention cells, and entrapment of powder in the interior region of the porous scaffold [99].

## 4. 3D Printed Polymers for Dental Applications

**Figure 4:**
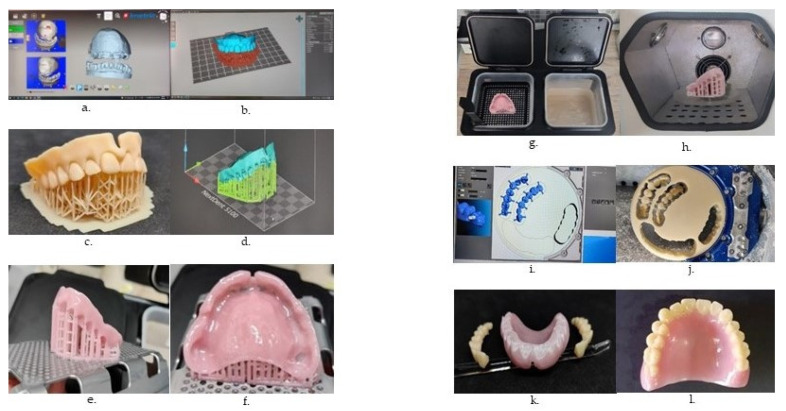
Workflow in 3D printing of a denture base, using vat photo-polymerization. (**a**) Obtaining the virtual model, by scanning; (**b**) digital design of the try-in; (**c**) 3D-printed try-in; (**d**) digital design of the denture base; (**e**,**f**) 3D-printed denture base; (**g**) cleaning with isopropanol; (**h**) post-polymerization with UV-light; (**i**) fabricating the artificial teeth by CAD/CAM milling; (**j**) attaching the teeth to the denture base; (**k**) polished, (**l**) completed denture.

Polymeric materials are currently the most common option for 3D printing in dentistry, used for manufacturing of fixed and removable dentures (Figure 4), and various other types of dental appliances, dental implants, and tissue structures [15].

Although various printable polymeric materials have been developed, their use in clinical applications is limited by their drawbacks [100]. However, the mechanical properties of the cured material can be tuned by adjusting the content of its different components, as well as the diluents, crosslinkers, or photoinitiators [101].

**Figure 5:**
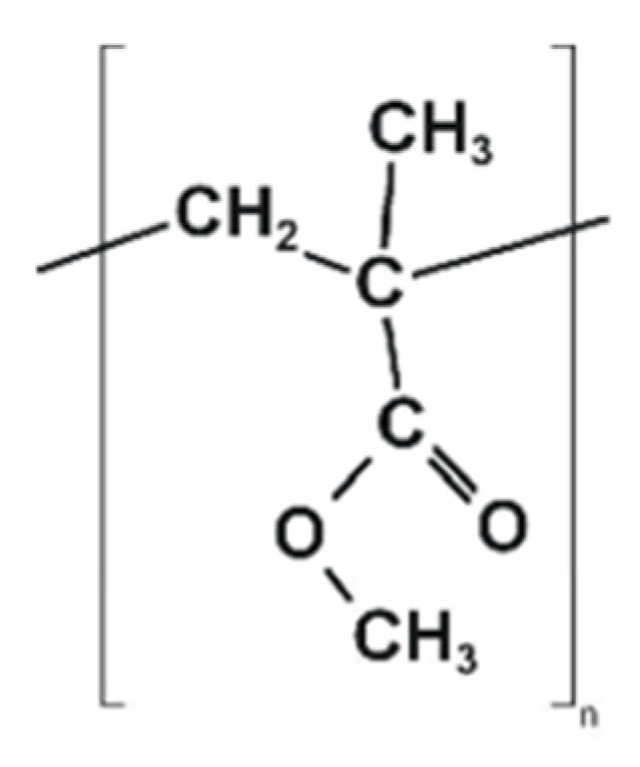
The chemical structure of PMMA.

Vinyl polymers are commonly used for 3D printing in dentistry, because of their tunable properties. PMMA (Figure 5), frequently used for SLA and SLS, has poor mechanical properties and a high shrinkage rate during light curing [102]. Mostly used for denture bases, it is lightweight, and stable in the oral environment. In order to enhance its antibacterial and mechanical properties, adding PEEK, TiO_2,_ SiO2, and Al2O3 has been attempted [102,103].

**Figure 6:**
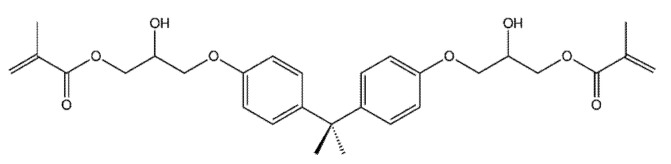
The chemical structure of Bis-GMA.

**Figure 7:**
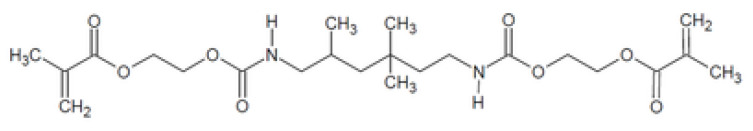
The chemical structure of UDMA.

**Figure 8:**
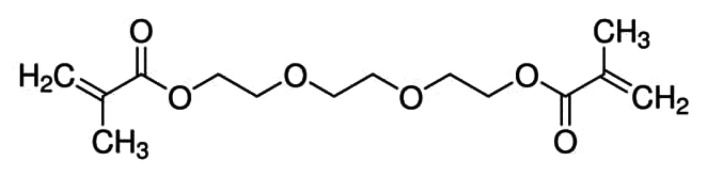
The chemical structure of TEGDMA.

Bisphenol A-glycidyl methacrylate (Bis-GMA) (Figure 6) and urethane dimethacrylate (UDMA) (Figure 7) based resins are being used as DLP materials, showing good mechanical properties, but because of their high molecular weights and viscosity, other components, as triethylene glycol dimethacrylate (TEGDMA) (Figure 8), need to be added, to reduce the viscosity and increase the rate of conversion [104].

**Figure 9:**
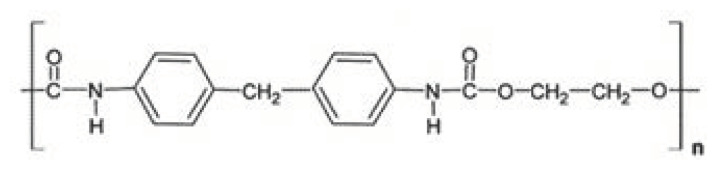
The chemical structure of PU.

PUs (Figure 9) have been widely used for biomedical applications, due to their wide wearability and biocompatibility [105]. Printable urethane acrylate-based resins with tunable mechanical properties for DLP exhibit high printability and good mechanical properties [100]. In order to decrease their viscosity and tune the mechanical properties, other components as poly (ethylene glycol) diacrylate (PEGDA) and propylene glycol (PPG) are to be added [101,106].

A wide range of thermoplastic polymers including PLA, ABS, PP, and PE are also being considered suitable for dental applications [10], PLA and ABS being more and more widely used as 3D-printing materials [107].

**Figure 10:**
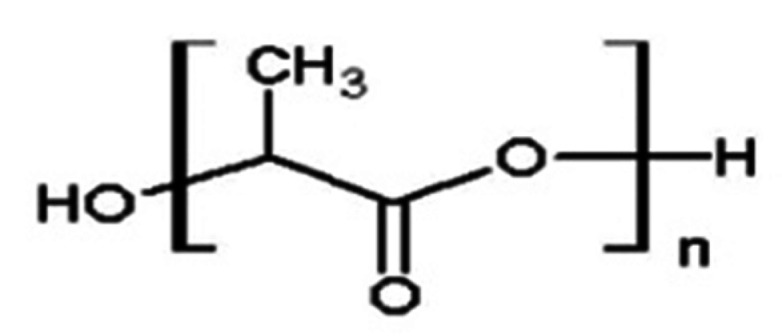
The chemical structure of PLA.

A biodegradable polyester, obtained from lactic acid, conventionally produced by sugar fermentation, PLA (Figure 10) stands as a low-cost, biocompatible, environment-friendly non-toxic material, with promising features such as processability, mechanical strength, and high impact resistance. PLA has been previously investigated as a material for 3D printing of provisional restorations [108], and drill guides for surgical insertion of dental implants [103]. The excellent processability enables its use in different 3D-printing methods, including FDM [103]. By means of surface modifications or combining it with other materials, it can be toughened and even used for dental implants [109].

**Figure 11:**
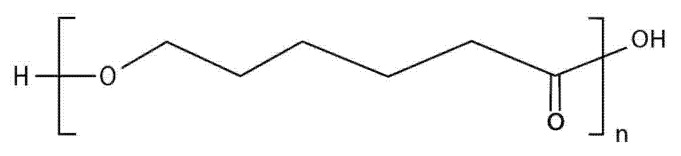
The chemical structure of PCL.

PCL (Figure 11) is another biodegradable and biocompatible polyester, characterized by high in vivo stability. Its synthesis involves the ring-opening polymerization of ε-caprolactone monomers in the presence of a catalyst. The low melting point recommends its use for printing techniques such as FDM [103].

**Figure 12:**
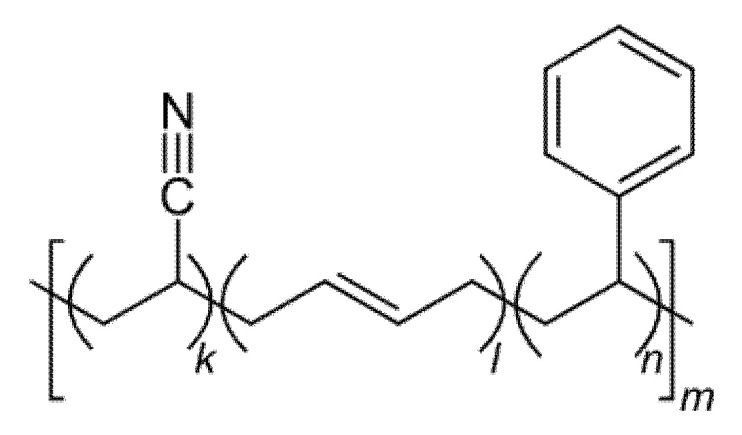
The chemical structure of ABS.

ABS (Figure 12), a thermoplastic polymer with an amorphous structure, obtained by polymerizing styrene and acrylonitrile, in the presence of polybutadiene, is considered less favorable than PLA, despite its high rigidity, and good impact resistance [110]. FDM and SLS are usually used for 3D printing of ABS [103]. Modification of ABS with different additives has been attempted, for instance silver nanoparticles, to enhance its antibacterial properties, when used as an implant material [110].

Both PLA and ABS are being used in their neat form, but numerous PLA and ABS-based composites are available [111].

When long-term stability is needed, non-degradable polymers such as PEEK are highly indicated.

**Figure 13:**
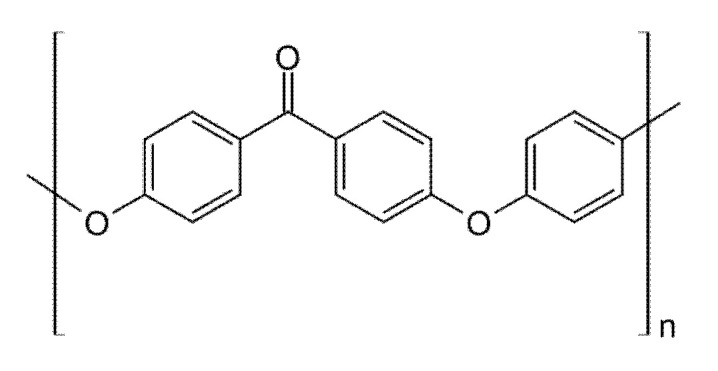
The chemical structure of PEEK.

PEEK (Figure 13) is a ketone-based semicrystalline, thermoplastic, high-performance polymer, with excellent mechanical and chemical resistance, high biocompatibility, insoluble, and lightweightness [112,113,114], successfully used for biomedical application, including fixed partial dentures, removable partial denture frameworks and clasps and implants [114,115]. 3D-printed PEEK, in the form of soluble epoxy-functionalized PEEK (ePEEK) and fenchone, is most often used with FDM, which requires increases in the nozzle and heating bed temperatures for PEEK materials [116]. However, as PEEK is bioinert, it lacks osseointegration, and it cannot support stress when used as an implant material [117]. To overcome its drawbacks and optimize its properties, surface modification by coating deposition or filler addition of bioactive hydroxyapatite, ceramic nanoparticles, and carbon fibers have been used [118].

Significant improvement in polymeric material properties, regardless of their form: liquid resin, powder or filament, combined with the advances in 3D-printing technology, currently enables manufacturing complex structures with various applications in dentistry [10].

## 5. Polymeric Bioinks

As alternatives to traditional inks, bioinks, which contain cells, have been developed for 3D bioprinting with clinical applications, including scaffolds, artificial organs, and tissues. Scaffolds are the key players in tissue engineering by inducing repair or initiate regenerative processes, with numerous applications in the oral area, such as management of periodontal diseases, or bone tissue regeneration [82]. Dental tissue engineering is also expected to regenerate damaged or lost components of tooth structure, including enamel, dentin, and pulp [119]. Scaffolds, as carriers for stem cells and growth factors, have a significant role in the regeneration of damaged or lost oral tissues [120]. Choosing the biomaterial with proper biological, chemical, and mechanical properties is considered of great importance in tissue engineering, scaffold biomaterials need to meet certain properties, including: biocompatibility, chemical stability, proper mechanical properties, absorbability, and degradability. They are also required to be non-toxic/non-carcinogenic, fit the targeted tissue, and stimulating the regenerative process, favor cell adhesion, differentiation, and proliferation [82].

Polymers are most attractive biomaterials for scaffolds because of their great tuning ability, including changes in composition, structure, and arrangement of constituents [119].

Polymers are currently indicated for a wide range of applications in hard and soft tissue regeneration in the field of dentistry and oral surgery, including tooth-germ, pulp–dentin, periodontal and salivary gland lesions, and bone regeneration [82].

Due to the required low fabrication temperature (below 37 °C), and the necessity to meet other important demands, only certain polymeric materials have been considered suitable for 3D bioprinting [121].

For example, despite their biocompatibility, thermoplastic PU’s and PCL have been used only as support structures, and discarded after printing, because of their high melting temperature (>90 °C), which would be harmful to cell cultures [122,123].

Some 3D-printable synthetic polymeric materials have been considered not suitable for 3D bioprinting because of their low cell affinity [124], or high toxicity [125], which can trigger adverse responses such as inflammation when used for clinical applications [126].

Polymers suitable for 3D bioprinting can be classified as: natural polymers, synthetic polymers, and hydrogels. Natural polymers, which include collagen, chitosan, gelatin, alginate, hyaluronic acid, decellularized extracellular matrix, and silk fibroin, are highly appreciated in the tissue engineering field, due to their biocompatibility, biodegradability, and bioactivity. They are mainly used as primary components for improving the biological properties of printed structures. Their drawbacks such as biodegradability, low mechanical stability, and immune reactivity to native cells or tissues, limit their use in tissue engineering. Their functional groups enable the functionalization for photo-polymerization [127,128,129].

Synthetic polymers include a wide choice of materials, such as PLA, PCL, PU, polyglycolic acid (PGA), polylactic-co-glycolic acid (PLGA), polyethylene glycol (PEG), ABS, PEEK, most frequently used as combinations [81,82,130]. They are generally characterized by robust mechanical features, higher processability, but lower biodegradability and biocompatibility because of the lack of cell adhesion sites [131]. Due to the biocompatibility issues, only a limited choice is suitable for biomedical applications. PCL is considered the most extensively studied polymer for 3D-bioprinted structures, because of easy availability, cost efficacy, and suitability for modification [132]. Other biodegradable polymers such as PLA, PGA, and their copolymers have also been widely considered for fabricating scaffolds for tissue engineering applications [133]. PLA has suitable mechanical properties, good processability, and adjustable degradation rate [134]. PGA is an aliphatic polyester which, due to its hydrolytic nature, shows a very fast degradation in aqueous solutions. Because its poor mechanical strength, it is mainly used for tuning the scaffold properties, and as a suture material [135].

Due to its non-degradable behavior, PEEK is not considered a choice for scaffold manufacture [136]. However, blending it with biodegradable polymers such as PGA and polyvinyl alcohol (PVA) enables producing degradable scaffolds [137], and further improvement can be achieved by adding nanofillers, such as hydroxyapatite [136]. ABS is another non-biodegradable polymer, suitable for blending [138]. Most of the synthetic polymers are subjected to chemical modifications with various reactive groups or blending with natural polymers, in order to gain controllable cell-mediated degradability and regulate cellular behavior such as spreading, proliferation, migration, and differentiation [139]. Their biocompatibility, printability, dimensional stability, and mechanical properties can be enhanced by adding bioactive nanofillers [140], such as hydroxyapatite, carbon nanotubes, graphene oxide, or magnetic nanoparticles [141,142].

Moreover, intelligent 3D-printed structures with dynamic features may be constructed by using polymers which have been formulated with stimuli-responsive, self-healable or shape-memorial components, enabling them to react to external environmental changes, such as pH, and temperature, impacting their biophysical properties [143].

Hydrogels are considered to be composite polymers, obtained by blending natural-natural or synthetic-natural polymeric materials, characterized by hydrophilic, three-dimensional networks [81]. They present the ability to absorb large quantities of water or biological fluids, and are an excellent choice for drug delivery systems, and scaffolds in tissue engineering, due to their biocompatibility, tunable biodegradability, proper mechanical strength, and porous structure. Moreover, bioactive agents may be incorporated in hydrogel-based bioinks [144].

The bioprintability of hydrogels highly depends on their rheological properties. Extrusion-based, droplet-based, and laser-based are suitable for bioprinting of hydrogel-based bioinks [144].

Depending on their chemical composition, hydrogels are responsive to various stimuli including heat, pH, light, and chemicals, and display the capability of combining with cells to engineer various tissues. However, due to their fragile nature, the applications of hydrogels are still limited [145,146].

Due to the progress in the field of bioinks and tissue engineering through multidisciplinary collaboration, their application in the dental area currently enables obtaining of scaffolds with desired structures and excellent performances, which facilitate tissue regeneration and eventually will finally result in whole tooth engineering [147].

## 6. 3D-Printing/Bioprinting Applications in Dentistry and Maxillofacial Surgery

3D printing is a useful tool both for clinical applications, as well as for educational purposes. For training purposes, different types of models, including hard and soft tissues, can be easily manufactured by 3D printing. Anatomic models of the orofacial region are being used in oral and maxillofacial surgery. 3D-printed teeth are equally used for teaching fixed prosthodontics, endodontics, and restorative dentistry. 3D-printed models also gradually replace document models in orthodontics [148,149,150,151].

Regarding 3D-printing applications in clinical dentistry, practically all disciplines are being currently covered. In oral and maxillifacial surgery, its indications include surgical guides, contour models for preoperative training, occlusal splints, and implants [152]. With the aid of surgical guides, preoperative planning for proper implant placing has become handy [153]. Surgical guides are equally suitable for single tooth to sinus lift, full mouth or zygomatic implants, reducing operation time and minimizing injuries [154,155,156].

Patient specific polymeric implants, used in cases of cranio-maxillofacial defects resulting from tumors, traumas, infections, or congenital deformities, have proven higher accuracy and better fit of the defect area [152,157,158].

Custom-made patient specific implants have also been introduced for reconstruction of temporo-mandibular joint or orthognathic surgery [159]. When coming to polymeric dental implants, 3D-printed PEEK has proven its suitability, so far [160].

Prosthodontics has probably benefited the most following the advancements in 3D printing. Both fixed and removable dentures may be easily manufactured using a complete digital flow, being characterized by precision and good fitting. Polymers are suitable for additive manufacturing of temporary crowns, denture bases, and artificial teeth. Even attempts for 3D printing polymeric permanent crowns and bridges have been made. Custom trays, patterns, try-ins, dental bites, and different types of models complete the applications of 3D-printed polymers in prosthodontics [161,162].

**Figure 14:**
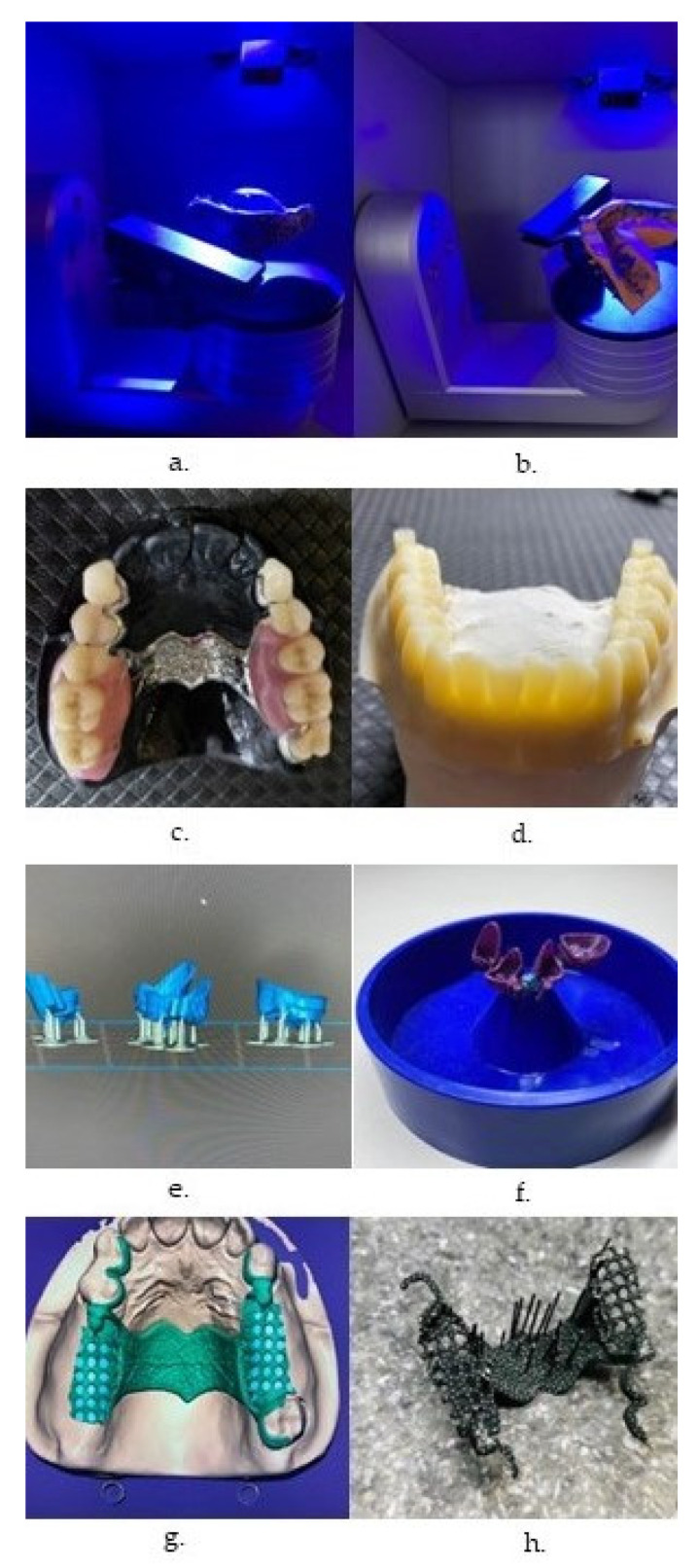
Combining different technologies, in manufacturing a removable partial denture. (**a**,**b**) Scanning the impressions; (**c**,**d**) 3D-printed models; (**e**) Digital design of the patterns; (**f**) 3D-printed copings pattern; (**g**) Digital design of the framework; (**h**) SLM 3D-printed metallic framework.

Different 3D-printing techniques may be combined in manufacturing certain types of prosthetic restorations (Figure 14)

Different types of orthodontic appliances may be manufactured by 3D printing, including aligners, custom brackets, occlusal splints, mouth guards, retainers, expanders, sleep apnea appliances, with better intraoral adaptation then regular ones [163,164,165,166].

In endodontics 3D printing has been involved in guided access cavity, guided root canal treatment and endo guided-surgical procedures, maneuvering obliterated pulp canals, and auto transplantation [167].

In periodontics, 3D-printed surgical guides are mainly used for esthetic surgery purposes. Additive manufacturing is increasingly being used to generate patient models, simulating the contours of the tissues and defects, including otherwise clinically unreachable areas [152].

3D printing has been even involved in restorative dentistry, intra coronal restorations obtained by additive manufacturing showing good accuracy and fit, comparable to conventional ones [168,169].

3D bioprinting in dentistry and maxillofacial surgery is currently involved in manufacturing soft and hard tissue structures for application in periodontal [170,171,172], periodontal ligament (PDL) [173,174], dental tissue [175,176,177,178], dental pulp [68,179,180], dentin [68,179,181], bone [182,183,184,185,186,187,188,189,190,191,192,193,194,195], and alveolar bone [182,183,196,197] regeneration, as well as head and neck cancer in vitro models [198] (Table 2).

The up to date research on dentoalveolar and maxillofacial tissue engineering has been mainly focused on bone tissue regeneration, of great importance in cases of hard tissue defects [199].

Bone augmentation is often necessary prior to implant placement, as insufficient bone structure results in poor support and consequent high rate of implant failure [200].

The destruction of periodontal ligament, involved in absorbing masticatory stresses results in reduced tooth support, increases tooth movement, and ultimately results in tooth loose due to periodontitis. 3D-bioprinted scaffolds, with incorporated stem cells, have proven efficient in regeneration of the periodontal ligament. Furthermore, soft tissue regeneration using 3D-printed scaffolds has been used in cases of gingival recession, involved in augmentation of the keratinized tissue surrounding teeth with periodontal defects [144].

3D bioprinting has been involved in attempting pulpal regeneration, as an alternative to conventional endodontic treatment, which includes removing the whole dental pulp tissue, and finally reduces the lifespan of the teeth [167,180].

Head and neck cancer in vitro models proved to be efficient in evaluating the standard-of-care therapeutics used in their treatment protocols [198].

Complex, hybrid, multilayered tissue structures have been attempted, the ultimate goal being to manufacture future additive for whole tooth [174].

## 7. 4D Printing and Bioprinting

The most significant limitation of 3D bioprinting is that it only considers the initial condition of the printed structure and not its subsequent changes over time. Tissue regeneration processes involve not only detailed 3D microarchitectures, and extracellular matrix compositions, but also specific tissue functions that are being obtained through dynamic conformation changes, due to internal or/and external stimuli. The later cannot be mimicked through 3D bioprinting [201].

**Figure 15:**
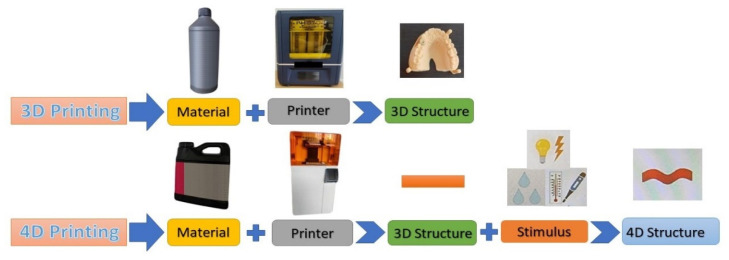
Comparative schematics of 3D- and 4D-printing technologies.

4D printing, incorporating “time” as the fourth dimension, was first demonstrated by Tibbits in 2014, enabling customized materials to change from one shape to another [202]. The capability of 4D-printed constructs “to transform over time, under different stimuli, and to adapt to the native microenvironments of defect areas, is expected to allow the creation of complicated structures with on-demand dynamically controllable shapes and functions” [203]. Being considered to be the future of tissue engineering, 4D bioprinting presents the possibility of constructing complex structures, which change their shape, color, and functionality under various physical, chemical, and biological stimuli [203], and has gained a lot of attention in the biomedical area, having numerous clinical applications [204,205,206] (Figure 15).

Sophisticated and programmable materials are being used for 4D printing, adding warm water, light, or heat to execute diverse functionalities [207,208]. The key in 4D printing is the intelligent material, characterized by its capability of shape change. Due to their thermo-mechanical characteristics and material qualities, intelligent materials are distinguished from ordinary 3D-printing materials by attributes of shape change [209], and can be categorized in materials which recover their original shape following a stimulus and materials which maintain their original shape and undergo morphology change in response to a stimulus [210].

Shape memory polymers and hydrogels are considered the most promising materials for 4D printing, due to a quicker reaction rate. Smart polymers are capable of remembering permanent shapes, owing to physical or chemical crosslinks. They present the capability to be deformed temporally, and return to their original shape under an external stimulus [211]. In tissue engineering, shape memory polymers have been considered for scaffolds manufacturing, whilst injectable hydrogels have proven effective for bone tissue engineering [203]. Because 4D bioprinting allows cell seeding only at the surface of smart polymers, rather than uniformly dispersing within their structure, cell-laden shape memory hydrogels, have been developed. The later can achieve reversible and sequential changes of their conditions and functions on demand [212].

In dentistry, 4D printing showed a good impact, as it can produce dynamic and adaptable materials, which have proven effective in the oral environment, under its continuously changing thermal and humidity conditions [213,214,215,216].

Its dental applications cover different specialties, including restorative dentistry, prosthodontics, orthodontics, implantology, and maxillo-facial surgery, enabling personalization of the therapeutic procedure.

In removable prosthetic dentistry, 4D-printed smart denture bases, characterized by similar elasticity and thermal properties as the oral tissues, can be obtained. By adjusting to the different types of applied forces, the materials used may be adapted to the stresses in the oral cavity, occlusion forces, eating, and drinking patterns included [217,218]. In case of special conditions, such as residual ridge resorption, including materials that compensate for bone loss has been attempted [213]. Other applications in prosthodontics include crown coping, and frameworks for partial dentures [219].

4D-printed fillings are most useful in inaccessible parts of the oral cavity where manipulating and maintaining is difficult. They may be designed to overcome current inconveniences such as dimensional changes, polymerization shrinkage, and microleakage, and adapt to predictable movements [219].

Intelligent, mobile, and detachable orthodontic appliances, with applications such as arch extension and bite-raising can be manufactured. Fixed orthodontic systems featuring intelligently moving bands and wires, prevent traumatic reactions in bones and periodontal ligaments, and subsequently improve long-time results [103].

4D-printed surgical guides, implants, and drug mouthguards are some of the latest advancements in implantology and maxillofacial surgery. Replacement of titanium alloys with shape memory polymers may result in improved biocompatibility and better osseointegration of implants. In temporomandibular joint problems, smart materials may be injected or surgically implanted [220].

4D-printed scaffolds, manufactured by utilizing organic components, including stem cells, proteins, and growth factors show an improved delivering capability to the target regions, thus enabling tissue to develop and surround them, initiating new tissue formation. Multilayer drug delivery systems, containing active substances, can be manufactured by 4D printing [219].

## 8. Concluding Remarks: Current Challenges and Future Perspectives

The application of 3D-printing technologies in dentistry allows mass customization, as well as digital workflow, with predictable lower cost and rapid turnaround times. Its potential to readily produce different types of prostheses, implants, as well as scaffolds with complex structures, will likely further enable involving tissue engineering in more dental disciplines [15]. The cumulative result of the advancements in both biomaterials and 3D-bioprinting technologies currently allows developing artificial tissues and organs with similar phenotype and function to the natural ones [221], and enable precise, reproducible, and large-scale fabrication of complex scaffolding systems with tunable architecture and physiomechanical properties [222].

With the aid of medical imaging, implants with patient- and damage-specific geometries, tuned porosity, size, and design may be manufactured even in case of large size bone grafts [223].

Integration of 3D-printing techniques with medical imaging technologies, such as computerized tomography, magnetic resonance imaging, laser digitizing or cone beam computed tomography, improves diagnosis, preoperative planning, quality and morphology of prosthetics and implants, and functional success of complex surgery [92,224].

Polymeric materials, in a continuous development, still remain the most frequent choice for these technologies [15]. Novel ink combinations enable further improving the printability and strength of the scaffolds [225,226]. Unfortunately, in certain cases, bone-like scaffolds showed inadequate reproducibility and patient-specificity. Their limited success in clinical applications, despite the promising in vitro and in vivo characteristics, is considered the result of the lack of proper osteoconductive cues in the bioink, biomaterial-related infections, and insufficient engraftment [227]. Continuous research in this area, provided the hyperelastic bone osteoregenerative bioink, composed of hydroxyapatite and PLGA, which allows printing of nanoparticle-functionalized bone scaffold systems with enhanced bacteriostatic properties and a complex, porous, and customized structure [228,229].

Novel techniques, such as Voxel printing, which actually allows programming the material and its viscosity, enable design freedom by manipulating both mechanical properties and color on a voxel-by-voxel basis, broadening the further applications of 3D-printing in the clinical research area [12].

The innovative 4D-printing strategies and smart materials provide great opportunities to fulfil the various challenges in dentistry, practically enabling a non-living object to, over time, modify its 3D form and behavior. They also present the potential for the manufacturing of complex multilayer tissue constructs, with doubtless advantages for future development of tissue engineering and its dental and maxillofacial applications [219]. 4D printing is expected to further boost the research into producing a whole tooth, capable to harmoniously integrate with the surrounding periodontium. However, predictable reconstruction of the whole tooth and its periodontal structures still remains challenging. 

## Figures and Tables

**Table 1:** Main characteristics of commonly used 3D-printing technologies for polymers in dentistry.

Characteristic	SLA	DLP	MJT	FDM
Type	vat photo-polymerization	vat photo-polymerization	material jetting	material extrusion
Resolution	high	high	high	low
Accuracy	medium	high	high	medium
Speed	medium	high	high	medium
Object size	scalable	scalable	scalable	scalable
Cost	medium	medium	high	low

**Table 2:** 3D bioprinting applications in dentistry and maxillofacial surgery.

Reference	Targeted Tissue	3D Bioprinting Technology	Bioink
[170]	Periodontal regeneration	Inkjet	GelMA + PEGDA
[171]	Periodontal regeneration	Extrusion	Collagen
[172]	Periodontal regeneration	Extrusion	GelMA
[173]	PDL regeneration	Extrusion	Collagen
[174]	PDL regeneration	Scaffold-free	-
[175]	Dental tissue regeneration	Extrusion	Gelatin + GelMA + HAc + glycerol
[176]	Dental tissue regeneration	Extrusion	Demineralized dentin matrix particles + fibrinogen + gelatin
[177]	Dental tissue regeneration	Extrusion	Poloxamer-407
[178]	Dental tissue regeneration	Extrusion	Collagen type 1 or dECM + β-TCP
[68]	Dentin/dental pulp regeneration	Extrusion	Alginate + dentin matrix
[179]	Dentin/dental pulp regeneration	Extrusion	Fibrinogen + gelatin + HAc + glycerol
[180]	Dental pulp regeneration	Inkjet	Collagen type 1 + agarose
[181]	Dentin regeneration	Extrusion	Calcium silicate + GelMA
[182]	Alveolar bone/bone regeneration	Extrusion	Gelatin + fibrinogen + HA + glycerol
[183]	Alveolar bone/bone regeneration	Extrusion	MeHAc + GelMA + HAc
[184]	Bone regeneration	Scaffold-free	-
[185]	Bone regeneration	Scaffold-free	-
[186]	Bone regeneration	Extrusion	ECM + AMP
[187]	Bone regeneration	Extrusion	Collagen + chitosan + β-glycer-ophosphate + nHA
[188]	Bone regeneration	Extrusion	Collagen + chitosan + β-glycerophosphate + nHA
[189]	Bone regeneration	Laser-based	Collagen type 1 + nHA
[190]	Bone regeneration	Laser-based	Collagen type 1
[191]	Bone regeneration	Laser-based	Collagen type 1
[192]	Bone regeneration	Laser-based	Collagen type 1 + TCP
[193]	Bone regeneration	SLA	GelMA
[194]	Bone regeneration	Extrusion	Sodium alginate + gelatin + nHA
[195]	Bone regeneration	Extrusion	Nanofibrillated cellulose + alginate
[196]	Alveolar bone in vitro model	SLA	GelMA + PEGDA
[197]	Alveolar bone regeneration	Inkjet	GelMA + PEGDA
[198]	Head and neck cancer in vitro model	Extrusion	Alginate + gelatin + dECM

GelMA: gelatin methacryloyl; PEGDA: poly(ethylene glycol) dimethacrylate; HAc: hyaluronic acid; Poloxamer-407: synthetic copolymer of poly(ethylene glycol) and poly(propylene glycol); dECM: decellularized extracellular matrix; TCP: tricalcium phosphate; MeHAc: methacrylated hyaluronic acid; ECM: extracellular matrix; AMP: amorphous magnesium phosphates; nHA: nano hydroxyapatite.

## Data Availability

Not applicable.
